# Patient Satisfaction With Telehealth Visits in Rural Compared With Urban Communities: Single-Center Study

**DOI:** 10.2196/85018

**Published:** 2026-06-24

**Authors:** Corrin Hepburn, Karolina Krawczyk, Cara Joyce, Jonah Rubin

**Affiliations:** 1 Division of Gastroenterology, Rush University Medical Center, 1620 W Harrison St, Chicago, IL, 60612, United States, 1 312 942 5000; 2 Division of Gastroenterology, Loyola University Medical Center, Maywood, IL, United States

**Keywords:** technology literacy, hepatology, patient experience, broadband, technological barriers

## Abstract

**Background:**

Studies performed in urban communities with access to technology suggest high patient satisfaction with telehealth. While virtual visits can increase the reach of clinical practice in rural communities, technological barriers may reduce patient satisfaction.

**Objective:**

This study aimed to compare satisfaction with telehealth visits between patients living in rural and urban communities.

**Methods:**

A telephone survey was developed and administered to hepatology patients seen at outpatient clinics from March 2020 through March 2021. Patient characteristics and survey responses were compared by urban and rural location as defined by the census tract based on zip code using univariable and multivariable logistic regression.

**Results:**

Of 400 patients, 164 (41%) completed the survey. Compared with urban patients, rural patients had twice the transportation time to clinic (mean 59, SD 35 vs mean 30, SD 15 min) and were more likely to cancel due to transportation issues (21/48, 46% vs 15/116, 13%). Rural patients reported less proficiency with technology and more technical difficulties, including an inability to log on to the portal or access the camera or microphone (35/48, 75% vs 29/116, 25%) and less comfort with their devices (26/48, 54% vs 10/116, 9%). Overall, urban patients were more likely to prefer telehealth (adjusted odds ratio 5.20, 95% CI 2.15‐13.7) and were more satisfied with telehealth vs in-person visits than rural patients (72/116, 62.1% vs 10/48, 20.8%).

**Conclusions:**

Rural patients reported more technical challenges with telehealth and more transportation issues than urban patients but favored in-person hepatology visits. Urban patients were more satisfied with telehealth visits compared with in-person visits. Research is needed to improve telehealth delivery and satisfaction for rural patients.

## Introduction

Telehealth provides an alternative to in-person appointments, and the number of these visits soared during the COVID-19 pandemic [1]. Potential benefits of telehealth include eliminating the need for transportation and reducing the amount of time associated with office visits, which can increase patient convenience and reduce health care costs. Previous research showed high patient satisfaction with telehealth visits, but most of these studies were performed in urban communities [1-3]. Despite the benefits, patients may experience difficulties with these visits related to internet connectivity and technological demands [1,4]. These difficulties are noted to be increased in patients with low technology literacy. Between 55% and 85% of households have broadband internet access, with lower rates in rural communities [5,6]. Our hepatology practice covers a wide geography with clinics throughout the state of Illinois, including patients in both rural and urban communities. Given the disparity in access to broadband in rural areas, our objective was to examine patient satisfaction with telehealth between rural and urban communities.

## Methods

### Ethical Considerations

This study was reviewed and approved by the institutional review board at Loyola University Medical Center (IRB#LU 214739; approval date October 21, 2021). All participants were adults and provided verbal informed consent prior to participation in the telephone survey. Participants were informed of the voluntary nature of the study, that refusal to participate would not affect their medical care, and that they could withdraw at any time. No protected health information was collected beyond data necessary for study objectives. Survey responses were deidentified and securely stored to maintain confidentiality, in accordance with institutional and ethical guidelines.

### Survey Development

A survey tool was developed to assess patient satisfaction with telehealth visits in addition to technology literacy and broadband internet access (Multimedia Appendix 1). Survey questions were identified from a literature search focusing on 4 main themes: demographic information, geographic information, telehealth literacy, and telehealth satisfaction. Telehealth literacy questions were developed to test similar concepts asked in prior validated questionnaires including the eHealth Literacy Scale [7] and the Mobile Device Proficiency Questionnaire [8]. Telehealth satisfaction questions were developed to test similar concepts asked in the Telehealth Usability Questionnaire and asked on a 5-point Likert scale [9].

Patients were approached for participation in this study if they had both a telehealth visit and an in-person appointment between March 2020 to March 2021, when our telehealth program was most active. We excluded patients who were liver transplant recipients and those with hepatocellular carcinoma, as their appointment criteria may require more frequent in-person visits with a multidisciplinary care team and therefore may bias the result. Patients were contacted by telephone by an investigator. Informed consent was obtained over the phone via a script. The survey was then read to these patients and responses were recorded in Research Electronic Data Capture (REDCap) software. For patients who were non–English speaking, a certified interpreter was used over the phone. Information on rural-urban commuting area code was calculated via the most recent US Department of Agriculture data by zip code for the 2010 census [10]. Demographic information was collected from the electronic medical record including age, gender, race, diagnosis, and zip code. Our primary outcome was patient satisfaction with the visit, measured on a 5-point Likert scale. Secondary outcomes included internet access, difficulties with telehealth use, device used, and preference for type of visit in the future.

### Statistical Analysis

Descriptive variables were compared using Student *t* tests (2-tailed) for continuous variables and chi-square or Fisher exact tests for nominal variables by location (urban vs rural as defined by census tract) and visit preference (in person or telehealth). Univariable and multivariable analyses with logistic regression models were used to determine odds ratios (ORs) for patient characteristics associated with telehealth preference. Certain questions in the survey used the Likert scale to assess characteristics associated with satisfaction with telehealth which were analyzed with proportional odds logistic regression models. Analyses were performed using the R programming language (version 4.4.1; R Foundation for Statistical Computing).

## Results

Of 400 patients who were contacted, 164 (41%) completed the survey. The mean age was 64 (SD 10) years, more than half were male participants (n=88, 53.7%), and three-quarters were non-Hispanic White (122/163, 74.8%). The most common causes of liver disease were alcohol (51/162, 31.5%), metabolic dysfunction–associated steatotic liver disease (49/162, 30.2%), and hepatitis C (36/162, 22.2%; Table 1).

**Table 1.** Patient demographics.

Characteristics	Overall (n=164)	Rural (n=48)	Urban (n=116)	*P* value
Age (y), mean (SD)^a^	64 (10)	65 (11)	63 (10)	.35
Sex, n (%)^c^				.86
Male	88 (53.7)	25 (52.1)	63 (54.3)	
Female	76 (46.3)	23 (47.9)	53 (45.7)	
Race, n (%)^a^				<.001
Black	14 (8.6)	0 (0)	14 (12.1)	
Hispanic	25 (15.3)	1 (2.1)	24 (20.7)	
Other	2 (1.2)	0 (0)	2 (1.7)	
White	122 (74.8)	46 (97.9)	76 (65.5)	
Diagnosis, n (%)^b^				.18
Alcohol-related liver disease	51 (31.5)	19 (40.4)	32 (27.8)	
Hepatitis C	36 (22.2)	12 (25.5)	24 (20.9)	
Metabolic dysfunction–associated steatotic liver disease	49 (30.2)	12 (25.5)	37 (32.2)	
Other	26 (16)	4 (8.5)	22 (19.1)	

a Data were available for 163 participants (overall),

b Data were available for 162 participants (overall),

c Data were available for 164 participants (overall)

Using univariate analysis, rural patients (n=48) compared with urban patients (n=116) had double the transportation time to the clinic (mean 59, SD 35 vs mean 30, SD 15 min; *P*<.001), were less likely to reliably attend appointments (36/48, 75% vs 110/116, 96%; *P*<.001), and were more likely to cancel visits due to transportation issues (21/48, 46% vs 15/116, 13%; *P*<.001; Table 2). Rural patients also reported more technical difficulties (35/48, 75% vs 29/116, 25%; *P*<.001), less comfort with their devices (26/48, 54% vs 10/116, 9%; *P*<.001), and less frequent computer use (14/48, 29% vs 78/116, 67%*; P*<.001). Despite these differences, similar proportions of rural and urban patients were willing to have telehealth visits in the future (38/48, 79% vs 100/116, 86%; *P*=.26).

**Table 2.** Patient responses to survey.

Characteristics	Overall (n=164)	Rural (n=48)	Urban (n=116)	*P* value
Time to clinic (min), mean (SD)^a^	39 (27)	59 (35)	30 (15)	<.001
Mode of transportation, n (%)				.56
Drive	161 (98.2)	48 (100)	113 (97.4)	
Public transportation	3 (1.8)	0 (0)	3 (2.6)	
Transportation, n (%)				.64
Drive yourself	117 (71.3)	33 (68.8)	84 (72.4)	
Depend on others	47 (28.7)	15 (31.3)	32 (27.6)	
Able to make appointments reliably, n (%)^a^	146 (89.6)	36 (75)	110 (95.7)	<.001
Had to cancel due to transportation issues, n (%)^b^	36 (22.2)	21 (45.7)	15 (12.9)	<.001
Internet connection, n (%)				<.001
Dial-up	1 (0.6)	0 (0)	1 (0.9)	
Broadband	122 (74.4)	26 (54.2)	96 (82.8)	
Satellite	7 (4.3)	4 (8.3)	3 (2.6)	
Other	1 (0.6)	0 (0)	1 (0.9)	
I do not know	33 (20.1)	18 (37.5)	15 (12.9)	
Owns a mobile phone, n (%)	162 (98.8)	48 (100)	114 (98.3)	>.99
Device with camera, n (%)^b^	154 (95.1)	43 (89.6)	111 (97.4)	.05
Device used for telehealth, n (%)				<.001
Computer	50 (30.5)	25 (52.1)	25 (21.6)	
Smartphone	70 (42.7)	14 (29.2)	56 (48.3)	
Tablet	44 (26.8)	9 (18.8)	35 (30.2)	
Technical difficulties, n (%)	65 (39.6)	36 (75)	29 (25)	<.001
How often uses a computer, n (%)				<.001
Daily	92 (56.1)	14 (29.2)	78 (67.2)	
Weekly	54 (32.9)	23 (47.9)	31 (26.7)	
Monthly	18 (11)	11 (22.9)	7 (6)	
Comfort with device, n (%)				<.001
Very uncomfortable	3 (1.8)	2 (4.2)	1 (0.9)	
Uncomfortable	33 (20.1)	24 (50)	9 (7.8)	
Neutral	25 (15.2)	12 (25)	13 (11.2)	
Comfortable	47 (28.7)	6 (12.5)	41 (35.3)	
Very comfortable	56 (34.1)	4 (8.3)	52 (44.8)	
Aware of myLoyola portal, n (%)^c^	148 (90.2)	40 (83.3)	108 (93.1)	.08
Communication with liver physician, n (%)^c^				<.001
Phone	87 (54)	37 (78.7)	50 (43.9)	
Email	49 (30.4)	0 (0)	49 (43)	
In person	25 (15.5)	10 (21.3)	15 (13.2)	
Communication with primary care provider, n (%)^a^				<.001
Phone	84 (51.5)	37 (77.1)	47 (40.9)	
Email	53 (32.5)	0 (0)	53 (46.1)	
In person	26 (16)	11 (22.9)	15 (13)	
Preferences for reminders, n (%)				<.001
Email	55 (33.5)	2 (4.2)	53 (45.7)	
Telephone	109 (66.5)	46 (95.8)	63 (54.3)	
Interested in new resources for telemedicine, n (%)^a^	131 (80.4)	37 (77.1)	94 (81.7)	.50
Easy to download app, n (%)	28 (17.1)	8 (16.7)	20 (17.2)	.93
How-to guide for accessing visits, n (%)	86 (52.4)	34 (70.8)	52 (44.8)	.002
App for symptoms to aid with medications, n (%)	64 (39)	8 (16.7)	56 (48.3)	<.001
Email reminders for myLoyola, n (%)	8 (4.9)	2 (4.2)	6 (5.2)	>.99
Important to have regular visits with liver physician, n (%)^a^	161 (98.8)	47 (100)	114 (98.3)	>.99
Understand more during virtual or in-person visits, n (%)				<.001
In person	59 (36)	28 (58.3)	31 (26.7)	
Virtual	3 (1.8)	0 (0)	3 (2.6)	
Equally between them	102 (62.2)	20 (41.7)	82 (70.7)	
Satisfaction with visit, n (%)				.97
Very unsatisfied	0 (0)	0 (0)	0 (0)	
Unsatisfied	2 (1.2)	0 (0)	2 (1.7)	
Neutral	3 (1.8)	1 (2.1)	2 (1.7)	
Satisfied	41 (25)	13 (27.1)	28 (24.1)	
Very satisfied	118 (72)	34 (70.8)	84 (72.4)	
Preference for visit type, n (%)				<.001
In person	88 (53.7)	39 (81.3)	49 (42.2)	
Virtual	76 (46.3)	9 (18.8)	67 (57.8)	
Receive same care during telehealth visit, n (%)^a^	126 (77.3)	31 (64.6)	95 (82.6)	.01
More satisfied with telehealth or in-person visits, n (%)				<.001
Telehealth	82 (50)	10 (20.8)	72 (62.1)	
In person	82 (50)	38 (79.2)	44 (37.9)	
Willing to have more telehealth visits in future, n (%)	138 (84.1)	38 (79.2)	100 (86.2)	.26

a Data were available for 163 participants (overall).

b Data were available for 162 participants (overall).

c Data were available for 161 participants (overall).

Multivariate analysis showed that 54% (88/164) of all participants reported a preference for in-person visits and 46% (76/164) for virtual visits. Furthermore, 81% (39/48) of rural patients preferred in-person visits compared with only 42% (49/116) of urban patients (*P*<.001). More urban patients were satisfied with telehealth visits compared with rural patients (72/116, 62% vs 10/48, 20%; *P*<.001). Both rural and urban patients had predominantly Medicare insurance (20/32, 63% vs 67/129, 51%); however, the proportion of Medicaid patients was greater in the urban population (26/129, 20% vs 4/32, 13%). Patients with Medicaid insurance preferred virtual visits to in-person visits (19/30, 63% vs 11/30, 37%, *P*=.03). Even with shorter travel times and fewer barriers to in-person visits, urban patients were more likely to prefer virtual visits compared with rural patients (OR 5.93, 95% CI 2.73‐14.1) and were more satisfied with telehealth visits compared with in-person visits (OR 6.22, 95%, CI 2.91‐14.3; Table 3). Other factors associated with patient satisfaction with telehealth visits included age (OR 0.96, 95% CI 0.93‐0.99) and comfort with their device (OR 6.08, 95% CI 3.75‐10.9). However, after adjusting for age, sex, location, and comfort with the device in a multivariable logistic regression, comfort with the device was the only significant predictor of patient satisfaction with telehealth visits compared with in-person visits (adjusted OR 5.80, 95% CI 3.30‐11.3; Table 3).

**Table 3.** Association of patient characteristics with telehealth satisfaction and preference.

	Prefers virtual visits, OR^a^ (95% CI)		More satisfied with telehealth than in person, OR (95% CI)		Satisfaction with virtual visit, OR (95% CI)	
	Unadjusted	Adjusted	Unadjusted	Adjusted	Unadjusted	Adjusted
Age (y)	0.96 (0.92‐0.99)	0.99 (0.95‐1.04)	0.96 (0.93‐0.99)	1.01 (0.96‐1.05)	1.00 (0.97‐1.04)	1.02 (0.98‐1.06)
Sex						
Female	1 (reference)	1 (reference)	1 (reference)	1 (reference)	1 (reference)	1 (reference)
Male	2.53 (1.35‐4.82)	1.77 (0.77‐4.09)	2.72 (1.46‐5.18)	2.04 (0.87‐4.88)	3.38 (1.68‐7.08)	2.84 (1.38‐6.05)
Comfort with device	5.36 (3.37‐9.33)	4.78 (2.81‐8.88)	6.08 (3.75‐10.9)	5.80 (3.30‐11.3)	1.39 (1.05‐1.86)	1.52 (1.03‐2.25)
Location						
Rural	1 (reference)	1 (reference)	1 (reference)	1 (reference)	1 (reference)	1 (reference)
Urban	5.93 (2.73‐14.1)	1.18 (0.36‐3.79)	6.22 (2.91‐14.3)	1.09 (0.32‐3.51)	1.06 (0.49‐2.18)	0.60 (0.23‐1.54)

a OR: odds ratio.

## Discussion

Rural patients in our study reported more difficulty attending in-person visits due to double the transportation time and were more likely to cancel these appointments due to transportation issues. Despite these challenges, rural patients reported higher satisfaction with in-person visits compared with urban patients. In contrast, rural patients experienced more technical difficulties during telehealth visits, reported less comfort with their devices, and used their smartphones less frequently. Notably, comfort with device use emerged as the strongest predictor of telehealth satisfaction, suggesting that lower technological literacy may underlie the lower preference for telehealth observed among rural patients.

Telehealth use has expanded rapidly, particularly during the post–COVID-19 pandemic era, due to its advantages in convenience, access, and timeliness [3,11,12]. Prior studies have demonstrated high satisfaction among both patients and health care providers, supporting telehealth as a viable alternative to in-person care [11,13,14]. Within the field of gastroenterology and hepatology, telehealth has been successfully integrated into inflammatory bowel disease clinics, with associated improvements in patient satisfaction and disease-specific quality of life [15]. In hepatology specifically, telehealth interventions have also been used to help support sleep, stress management, and nutrition [16-18]. Within academic gastroenterology and hepatology practices, factors associated with higher satisfaction have included younger age, race, and type of visit [14]. However, broader socioeconomic factors are increasingly recognized as important contributors [19,20]. Prior reviews have demonstrated that income and education level are key determinants of telehealth accessibility and are closely linked to lower electronic health literacy [3,21,22]. Consistent with this literature, our study found that preference for virtual visits was associated with younger age, urban location, smartphone or tablet use, and greater comfort with technology. We also observed significantly lower telehealth satisfaction among rural patients, which appeared to be primarily driven by reduced comfort with devices and lower technology literacy. Although age alone was not independently associated with visit-type preference in multivariable analysis (OR 0.97; *P*=.20), patients with Medicaid insurance, used as a proxy for income, were more likely to prefer virtual visits (*P*=.03), highlighting the complex interaction between socioeconomic factors and telehealth preferences.

Telehealth has been shown to increase access to care through methods of improving availability and affordability [23]. However, persistent challenges remain, particularly related to infrastructure limitations such as broadband access and gaps in technology literacy. Our results show that rural patients are disproportionately affected by both barriers. While the Infrastructure Act of 2021 promised US $65 billion toward improving broadband access, addressing connectivity alone may be insufficient. Targeted efforts to improve technology literacy are likely necessary to fully realize the benefits of telehealth in rural populations.

To better understand potential solutions, our survey asked patients to identify resources that might help them with their visits in the future. Proposed interventions included a step-by-step “how-to” guide for accessing visits, a loaned tablet with a preloaded telehealth app, and a hospital-based app for tracking weight or medication management. A total of 80% (131/164) of patients expressed interest in additional resources, with the most endorsed intervention being a “how-to” guide focusing on accessing visits. These findings suggest that relatively low-cost educational interventions may meaningfully improve telehealth satisfaction. Future studies should focus on implementing such interventions, particularly in rural communities, to assess whether targeted technology training and assistance can help improve health care access and patient satisfaction.

The Veterans Affairs (VA) health care system was an early adopter of telehealth and provides a useful model. Through initiatives led by the VA Office of Connected Care, established in 2015, the VA has addressed common telehealth barriers by providing loaned devices and offering structured support to improve technology literacy [24]. These investments in telehealth have allowed the VA to reach a broader patient population [25]. Whether other health care systems and private insurers will adopt similar programs to address these barriers remains an important area for study.

Several limitations should be considered when interpreting our findings. This was a single-center study conducted during the COVID-19 pandemic, and some patients may have favored telehealth visits due to concerns about infection risk with in-person visits. Although our survey was not validated externally, it was based on prior studies and reviewed by our hepatology colleagues and statistician to ensure appropriate content. Visit types were not stratified by routine follow-ups and acute visits, which may have played a role in satisfaction. In addition, patient-physician relationships may have confounded satisfaction ratings, as patients’ comfort with a physician may have biased their opinion on visit type. Furthermore, given the complexity of our patient population, those who answered the survey may have had higher technology literacy at baseline, potentially limiting generalizability to the broader hepatology patient population. Finally, our study did not address health care provider burden or perceived quality of care [26]. Health care provider concerns regarding limitations of physical examinations during telehealth visits were not evaluated, nor were objective clinical outcomes assessed. Future studies should incorporate both patient- and health care provider–reported outcomes to better characterize the impact of telehealth on quality of care.

In conclusion, this study demonstrates lower satisfaction with telehealth among rural patients compared with urban patients. Our findings suggest that efforts to improve broadband access, provide appropriate devices, and enhance telehealth literacy may increase satisfaction and reduce barriers to care, including travel time and associated costs, for patients in rural areas.

## Supplementary material

10.2196/85018 Multimedia Appendix 1 Supplemental materials including survey questionnaire and other data tables.

## Abbreviations

OR: odds ratio
REDCap: Research Electronic Data Capture
VA: Veterans Affairs
