# Supplementary material for: Patient Satisfaction With Telehealth Visits in Rural Compared With Urban Communities: Single-Center Study
Source: JMIR Aging. 2026 Jun 24;9:e85018. doi: 10.2196/85018 (PMC13293599; doi:10.2196/85018)
Supplement: Multimedia Appendix 1 [file aging-v9-e85018-s001.docx]

**S1: Survey Tool**

1. How long does it take you to travel to Liver Clinic from home?
2. How do you get to your Liver Clinic appointments?
   1. Drive, public transportation, taxi, walk, other
3. Do you drive yourself to your liver appointments or depend on others to drive you?
4. Are you able to make your liver appointments reliably?
5. Have you had to cancel due to transportation issues?
6. Do you have an internet provider, if so, which one?
   1. Xfinity, AT&T, Earthlink, RCN, WOW, CenturyLink, other
7. How do you connect to the internet?
   1. Dial up, broadband, satellite, other, I don’t know
8. Do you own a mobile phone?
9. Does your computer, tablet, or smartphone have a camera?
10. What device do you use for your telehealth visits?
11. Have you had technical difficulties with your telehealth visits?
12. How often do you use your tablet or computer?
    1. Daily, weekly, monthly
13. On a scale of 1-5, how comfortable are you with using your computer or smartphone?
    1. 1 = very uncomfortable, 5 = very comfortable
14. Are you aware of the online MyLoyola portal?
15. How do you communicate with your liver doctor?
16. How do you communicate with your PCP?
17. Do you prefer email or telephone reminders with regards to your appointments?
18. Would you be interested in future resources to aid in your telemedicine experience?
    1. If yes – which of these would be the most helpful?
       1. A how to guide, a take home tablet, hospital app to document weight
19. Do you feel it is important that you have regular visits with your Liver Doctor?
20. Do you feel you understand your condition more during the virtual or in person visit?
21. On a scale of 1-5, how satisfied are you with your above visit?
    1. 1 = very unsatisfied, 5 = very satisfied
22. Would you prefer in person or virtual visits?
23. Which do you prefer, an in person visit for a new condition, old condition, or routine condition?
24. Do you prefer telehealth visits for new condition, routine condition, or old condition
25. Do you feel you receive the same care with a telehealth visit that you would with an in person visit?
26. Are you more satisfied with your telehealth visit or your in person visit?

**S2: Insurance Status by Location**

|  | Urban | Rural |
| --- | --- | --- |
| Medicaid (n=30) | 26 | 4 |
| Medicare (n=87) | 67 | 20 |
| Private (n=44) | 36 | 8 |

**S3: Preference for visit type by insurance status**

|  | Preference for in person visit | Preference for telehealth |
| --- | --- | --- |
| Medicaid | 11 | 19 |
| Medicare | 54 | 33 |
| private | 20 | 24 |

**S4: Patient characteristics and survey responses by visit type preference**

| Region | **N** | **Overall**, N = 164 | **In person**, N = 88 | **Virtual**, N = 76 | **p-value** |
| --- | --- | --- | --- | --- | --- |
| Region, n (%) | 164 |  |  |  | <0.001 |
| Rural |  | 48 (29.3%) | 39 (44.3%) | 9 (11.8%) |  |
| Urban |  | 116 (70.7%) | 49 (55.7%) | 67 (88.2%) |  |
| Age, Mean (SD) | 163 | 64 (10) | 66 (10) | 62 (10) | 0.006 |
| Sex, n (%) | 164 |  |  |  | 0.005 |
| Male |  | 88 (53.7%) | 38 (43.2%) | 50 (65.8%) |  |
| Female |  | 76 (46.3%) | 50 (56.8%) | 26 (34.2%) |  |
| Race, n (%) | 163 |  |  |  | 0.22 |
| Black |  | 14 (8.6%) | 5 (5.7%) | 9 (11.8%) |  |
| Hispanic |  | 25 (15.3%) | 13 (14.9%) | 12 (15.8%) |  |
| Other |  | 2 (1.2%) | 0 (0.0%) | 2 (2.6%) |  |
| White |  | 122 (74.8%) | 69 (79.3%) | 53 (69.7%) |  |
| Diagnosis, n (%) | 162 |  |  |  | 0.027 |
| Alcoholic |  | 51 (31.5%) | 26 (29.5%) | 25 (33.8%) |  |
| HCV |  | 36 (22.2%) | 14 (15.9%) | 22 (29.7%) |  |
| NASH |  | 49 (30.2%) | 28 (31.8%) | 21 (28.4%) |  |
| Other |  | 26 (16.0%) | 20 (22.7%) | 6 (8.1%) |  |
| Time to clinic (minutes), Mean (SD) | 163 | 39 (27) | 43 (28) | 33 (25) | 0.018 |
| Mode of transportation, n (%) | 164 |  |  |  | 0.10 |
| Drive |  | 161 (98.2%) | 88 (100.0%) | 73 (96.1%) |  |
| Public Transportation |  | 3 (1.8%) | 0 (0.0%) | 3 (3.9%) |  |
| Transportation, n (%) | 164 |  |  |  | 0.002 |
| drive yourself |  | 117 (71.3%) | 54 (61.4%) | 63 (82.9%) |  |
| depend on others |  | 47 (28.7%) | 34 (38.6%) | 13 (17.1%) |  |
| Able to make appointments reliably, n (%) | 163 | 146 (89.6%) | 77 (87.5%) | 69 (92.0%) | 0.35 |
| Had to cancel due to transportation issues, n (%) | 162 | 36 (22.2%) | 24 (27.9%) | 12 (15.8%) | 0.064 |
| Internet connection, n (%) | 164 |  |  |  | <0.001 |
| Dial up |  | 1 (0.6%) | 0 (0.0%) | 1 (1.3%) |  |
| Broadband |  | 122 (74.4%) | 56 (63.6%) | 66 (86.8%) |  |
| Satellite |  | 7 (4.3%) | 6 (6.8%) | 1 (1.3%) |  |
| Other |  | 1 (0.6%) | 0 (0.0%) | 1 (1.3%) |  |
| I don’t know |  | 33 (20.1%) | 26 (29.5%) | 7 (9.2%) |  |
| Owns a mobile phone, n (%) | 164 | 162 (98.8%) | 86 (97.7%) | 76 (100.0%) | 0.50 |
| Device with camera, n (%) | 162 | 154 (95.1%) | 81 (93.1%) | 73 (97.3%) | 0.29 |
| Device used for telehealth, n (%) | 164 |  |  |  | <0.001 |
| Computer |  | 50 (30.5%) | 43 (48.9%) | 7 (9.2%) |  |
| Smartphone |  | 70 (42.7%) | 25 (28.4%) | 45 (59.2%) |  |
| Tablet |  | 44 (26.8%) | 20 (22.7%) | 24 (31.6%) |  |
| Technical difficulties, n (%) | 164 | 65 (39.6%) | 59 (67.0%) | 6 (7.9%) | <0.001 |
| How often uses computer, n (%) | 164 |  |  |  | <0.001 |
| Daily |  | 92 (56.1%) | 36 (40.9%) | 56 (73.7%) |  |
| Weekly |  | 54 (32.9%) | 35 (39.8%) | 19 (25.0%) |  |
| Monthly |  | 18 (11.0%) | 17 (19.3%) | 1 (1.3%) |  |
| Comfort with device, n (%) | 164 |  |  |  | <0.001 |
| very uncomfortable |  | 3 (1.8%) | 3 (3.4%) | 0 (0.0%) |  |
| uncomfortable |  | 33 (20.1%) | 30 (34.1%) | 3 (3.9%) |  |
| neutral |  | 25 (15.2%) | 24 (27.3%) | 1 (1.3%) |  |
| comfortable |  | 47 (28.7%) | 23 (26.1%) | 24 (31.6%) |  |
| very comfortable |  | 56 (34.1%) | 8 (9.1%) | 48 (63.2%) |  |
| Aware of myLoyola portal, n (%) | 164 | 148 (90.2%) | 74 (84.1%) | 74 (97.4%) | 0.004 |
| Communication with liver doctor, n (%) | 161 |  |  |  | <0.001 |
| phone |  | 87 (54.0%) | 52 (60.5%) | 35 (46.7%) |  |
| email |  | 49 (30.4%) | 9 (10.5%) | 40 (53.3%) |  |
| in person |  | 25 (15.5%) | 25 (29.1%) | 0 (0.0%) |  |
| Communication with PCP, n (%) | 163 |  |  |  | <0.001 |
| Phone |  | 84 (51.5%) | 51 (58.6%) | 33 (43.4%) |  |
| Email |  | 53 (32.5%) | 10 (11.5%) | 43 (56.6%) |  |
| In person |  | 26 (16.0%) | 26 (29.9%) | 0 (0.0%) |  |
| Preferences for reminders, n (%) | 164 |  |  |  | <0.001 |
| email |  | 55 (33.5%) | 13 (14.8%) | 42 (55.3%) |  |
| telephone |  | 109 (66.5%) | 75 (85.2%) | 34 (44.7%) |  |
| Interested in new resources for telemedicine, n (%) | 163 | 131 (80.4%) | 58 (66.7%) | 73 (96.1%) | <0.001 |
| Easy to download app, n (%) | 164 | 28 (17.1%) | 13 (14.8%) | 15 (19.7%) | 0.40 |
| How to guide for accessing visits, n (%) | 164 | 86 (52.4%) | 52 (59.1%) | 34 (44.7%) | 0.066 |
| App for symptoms to aid with meds, n (%) | 164 | 64 (39.0%) | 13 (14.8%) | 51 (67.1%) | <0.001 |
| Email reminders for myLoyola, n (%) | 164 | 8 (4.9%) | 2 (2.3%) | 6 (7.9%) | 0.15 |
| Important to have regular visits with liver doctor, n (%) | 163 | 161 (98.8%) | 86 (97.7%) | 75 (100.0%) | 0.50 |
| Understand more during virtual or in person visits, n (%) | 164 |  |  |  | <0.001 |
| In person |  | 59 (36.0%) | 56 (63.6%) | 3 (3.9%) |  |
| Virtual |  | 3 (1.8%) | 0 (0.0%) | 3 (3.9%) |  |
| Equally between them |  | 102 (62.2%) | 32 (36.4%) | 70 (92.1%) |  |
| Satisfaction with visit, n (%) | 164 |  |  |  | <0.001 |
| very unsatisfied |  | 0 (0.0%) | 0 (0.0%) | 0 (0.0%) |  |
| unsatisfied |  | 2 (1.2%) | 2 (2.3%) | 0 (0.0%) |  |
| neutral |  | 3 (1.8%) | 3 (3.4%) | 0 (0.0%) |  |
| satisfied |  | 41 (25.0%) | 31 (35.2%) | 10 (13.2%) |  |
| very satisfied |  | 118 (72.0%) | 52 (59.1%) | 66 (86.8%) |  |
| Receive same care during telehealth visit, n (%) | 163 | 126 (77.3%) | 53 (60.2%) | 73 (97.3%) | <0.001 |
| More satisfied with telehealth or in person, n (%) | 164 |  |  |  | <0.001 |
| telehealth |  | 82 (50.0%) | 10 (11.4%) | 72 (94.7%) |  |
| in person |  | 82 (50.0%) | 78 (88.6%) | 4 (5.3%) |  |
| Willing to have more telehealth visits in future, n (%) | 164 | 138 (84.1%) | 62 (70.5%) | 76 (100.0%) | <0.001 |

**S5: Ethical Statement**
This study was reviewed and approved by the Institutional Review Board at Loyola University Medical Center, IRB#LU 214739, approval date 10/21/2021. All participants were adults and provided verbal informed consent prior to participation in the telephone survey. Participants were informed of the voluntary nature of the study, that refusal to participate would not affect their medical care, and that they could withdraw at any time. No protected health information was collected beyond data necessary for study objectives. Survey responses were de-identified and securely stored to maintain confidentiality, in accordance with institutional and ethical guidelines.

**S6: Patient demographics by survey participation**

| Region | **Did not complete survey, N = 236** | **Completed survey, N = 164** | **p-value** |
| --- | --- | --- | --- |
| Age, Mean (SD) [n=399] | 64 (11) | 64 (10) | 0.77 |
| Sex, n (%) |  |  | 0.22 |
| Male | 112 (47.5%) | 88 (53.7%) |  |
| Female | 124 (52.5%) | 76 (46.3%) |  |
| Race, n (%) [n=398] |  |  | 0.22 |
| Black | 16 (6.8%) | 14 (8.6%) |  |
| Hispanic | 41 (17.4%) | 25 (15.3%) |  |
| Other | 11 (4.7%) | 2 (1.2%) |  |
| White | 167 (71.1%) | 122 (74.8%) |  |
| Diagnosis, n (%) [n=398] |  |  | 0.27 |
| Alcoholic | 67 (28.4%) | 51 (31.5%) |  |
| HCV | 38 (16.1%) | 36 (22.2%) |  |
| NASH | 88 (37.3%) | 49 (30.2%) |  |
| Other | 43 (18.2%) | 26 (16.0%) |  |
| Region, n (%) [n=396] |  |  | <0.001 |
| Rural | 35 (15.1%) | 48 (29.3%) |  |
| Urban | 197 (84.9%) | 116 (70.7%) |  |
